# Exposure to High Fluoride Drinking Water and Risk of Dental Fluorosis in Estonia

**DOI:** 10.3390/ijerph6020710

**Published:** 2009-02-16

**Authors:** Ene Indermitte, Astrid Saava, Enn Karro

**Affiliations:** 1 Department of Public Health, University of Tartu, Ravila 19, 50411 Tartu, Estonia; E-Mail: astrid.saava@ut.ee; 2 Department of Geology, University of Tartu, Vanemuise 46, 51014 Tartu, Estonia; E-Mail: enn.karro@ut.ee

**Keywords:** Drinking water, fluoride, exposure, dental fluorosis, Estonia

## Abstract

The purpose of this study was to assess exposure to drinking water fluoride and evaluate the risk of dental fluorosis among the Estonian population. The study covered all 15 counties in Estonia and 93.7% of population that has access to public water supplies. In Estonia groundwater is the main source for public water supply systems in most towns and rural settlements. The content of natural fluoride in water ranges from 0.01 to 7.20 mg/L. The exposure to different fluoride levels was assessed by linking data from previous studies on drinking water quality with databases of the Health Protection Inspectorate on water suppliers and the number of water consumers in water supply systems. Exposure assessment showed that 4% of the study population had excessive exposure to fluoride, mainly in small public water supplies in western and central Estonia, where the Silurian-Ordovician aquifer system is the only source of drinking water. There is a strong correlation between natural fluoride levels and the prevalence of dental fluorosis. Risk of dental fluorosis was calculated to different fluoride exposure levels over 1.5 mg/L.

## 1. Introduction

Fluoride is known to have both beneficial and adverse effects on humans, depending on the total intake. Drinking water is usually, but not always, the main source of fluoride and fluoride is sometimes added to public water supplies to help prevent dental caries. This paper considers exposure to natural fluoride through public drinking water supplies in Estonia in 2004. The WHO health-based guideline value for drinking water [1], which is also the basis of the value in the EC Drinking Water Directive transposed into Estonian Law by the Estonian regulations [2], is 1.5 mg/L.

Naturally occurring high fluoride levels in groundwater is a complicated issue for drinking water providers in many regions of the world. They are faced with dilemma that deep groundwater from drilled wells is bacteriologically safe but is often not suitable because of the presence of excess naturally occurring chemicals such as fluorides, whereas surface water and water from traditional shallow dug wells have lower fluoride contents, but can be contaminated by faecal material.

High fluoride groundwater occurs in many areas of the world. The majority of epidemiological studies about fluoride levels in drinking water and their health effects have been carried out in developing countries in Asia, Africa and Middle-East, where there are significantly elevated concentrations of fluoride [3]. Less data are available for the European region. Fluoride can be beneficial in helping to prevent dental caries at drinking water concentrations of about 1 mg/L but it has also been shown to cause dental mottling and adverse effects on bone, including increased risk of fracture at concentrations in excess of 1.5 mg/L, with the risk gradually increasing with the total intake of fluoride [4,5].

Groundwater is the main drinking water source for most of Estonia’s towns and rural settlements. Depending on the hydro-geological conditions five different aquifer systems are exploited. Only in two towns, where groundwater resources are limited, is surface water used. Studies on occurrence and distribution of fluoride in groundwater have shown high fluoride contents of some aquifer systems, especially in the western part of country [6–8]. The highest fluoride concentrations are detected in areas where Silurian and Ordovician limestones and dolomites form the Silurian-Ordovician aquifer system. However, nobody has performed an exposure assessment among the Estonian population. On the other hand, some studies of the adverse effects of fluoride exposure have been carried out in Estonia [9,10].

The present study was undertaken to assess the distribution of high fluoride drinking water over the whole of Estonia, the extent of population exposure to different levels of fluoride and the risk of dental fluorosis. These results should be taken into account when developing strategies for the supply of safe drinking water.

## 2. Materials and Methods

### 2.1. Study Area

Estonia is the smallest Baltic country, with an area of 45,227 km^2^ and population of 1,351 million (01.01.2004) people. Administratively Estonia is divided into 15 counties. Surface water in two towns (the capital Tallinn and Narva) and groundwater from several aquifers accessed by drilled wells in other towns and rural settlements are the main sources of drinking water. Five aquifer systems are exploited, depending on location. In most cases, groundwater is used directly for drinking purposes without any treatment.

**Figure 1.**
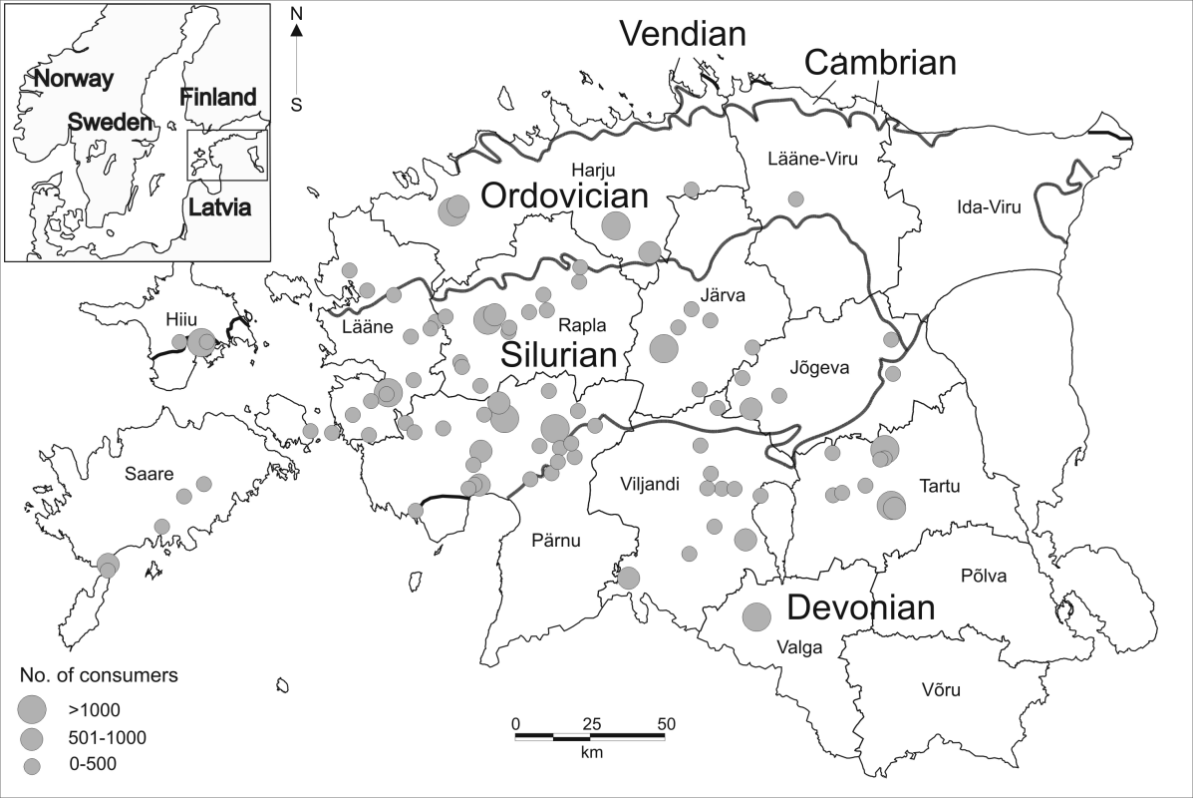
Map of study area, location of water supply systems with high fluoride content (>1.5 mg/L) by number of water consumers.

Estonia is situated in the north-western part of the East-European Platform. Its sedimentary beds, lying on the southern slope of the Baltic Shield, are gradually sloping by 2 4 metres per kilometre towards the south. The crystalline Paleoproterozoic basement is overlaid by Neoproterozoic (Vendian) and Palaeozoic (Cambrian, Ordovician, Silurian and Devonian) sedimentary rocks (Figure 1) covered by Quaternary deposits. The thickness of the sedimentary rocks is increasing from the north (150 m) to the south (700 m). Southwards, in Latvia, there are crystalline basement rocks lying at a depth of 500–1,800 metres, but towards the north, in Finland, the basement rocks outcrop on the land surface and the sedimentary rocks are practically missing.

Hydrogeologically, Estonian sedimentary rocks form a typical artesian basin, where five aquifer systems (Middle-Devonian, Middle-Lower-Devonian, Silurian-Ordovician, Ordovician-Cambrian and Cambrian-Vendian) are isolated from each other by impervious beds. Quaternary deposits, consisting predominantly of glacial till and glaciolacustrine sandy loam, form the uppermost aquifer system, which is used as a drinking water source mostly in private households.

The Middle-Devonian and Middle-Lower-Devonian aquifer systems are the main source of public water supply in southern Estonia. Groundwater is abstracted from terrigeneous material: sand- and siltstones with interlayers of clayey and dolomitized sandstone. The Silurian-Ordovician aquifer system consists of diverse limestone and dolomite with clayey interlayers. The upper portion of the water bearing rocks, with a thickness of 30 m, is intensively fractured and cavernous. The aquifer system is an important drinking water source in western and central Estonia. The Ordovician-Cambrian aquifer system is present in most of Estonia, except the islands of the West-Estonian Archipelago. The aquifer system consists of fine-grained sandstones and siltstones with total thickness of 60 m and is exploited in the northern and central part of the country. The deepest economically important Cambrian-Vendian aquifer system consists of sand- and siltstones with interlayers of clay. In southern and central Estonia the aquifer system contains relict saline groundwater. Cambrian-Vendian aquifer system is, besides surface water, the major source of public water supplies in northern Estonia, where groundwater is fresh.

### 2.2. Methodology

The present study links available data of water quality from previous studies [8, 11] with data on water consumers. Data on access of population to water supplies was obtained from the Estonian Health Protection Inspectorate database, which includes information about public water suppliers, number of water consumers, source of water and amount of consumed water. All drinking water supplies serving at least 100 consumers were included into the study. Fluoride concentration in drinking water was categorized into three groups: low-fluoride water (up to 0.50 mg/L), medium-fluoride water (0.51–1.50 mg/L) and high-fluoride water (over 1.50 mg/L). For health risk assessment purposes the population exposed to excessive fluoride (over 1.5 mg/L) was divided into four exposure categories by content of fluoride in drinking water:

1. 1.51–2.0 mg/L
2. 2.1–3.0 mg/L
3. 3.1–4.0 mg/L
4. > 4.0 mg/L

The risk estimation was performed using original data from two previous studies carried out in Estonia on the relationship between dental fluorosis and drinking water fluoride content. In the first study [9], 7–15 years-old schoolchildren were studied in eight settlements differing by fluoride content in the drinking water. The clinical examinations were performed by experienced dentists. The diagnosis of dental fluorosis followed the criteria of Dean [12]. The second study [10] was part of the all-Estonian survey of dental health of 12-years-old schoolchildren, conducted according to the uniformed methodology of the World Health Organisation [13]. The sub-sample was selected from town of Tartu, where the fluoride content in drinking water varied significantly between regions. Schoolchildren were localized according to their home address and their match to drinking water regions with different fluoride levels was determined. The clinical intra-oral examinations were conducted at the schools by a trained dentist. Dental fluorosis was assessed on vestibular, occlusal and lingual surfaces. Any white flecks, fine white and brown lines in the enamel were registered as a mild degree of fluorosis. Very chalky, opaque enamel, mottling and loss of portions of the outer enamel were diagnosed as severe fluorosis [14]. In both studies, only children who had reported a lifelong residence in a region were included in the risk assessment. The total sample size was 2,627 subjects. The risk of dental fluorosis was expressed as the odds ratio of the disease (OR). Data were analyzed using the Statistical Package for Social Sciences (SPSS, version 11.0).

## 3. Results

The results of this study provide a first assessment of human exposure to fluorides by drinking water and risk of dental fluorosis.

### 3.1. Distribution of High Fluoride Drinking Water in Estonia

The Estonian population is well provided with public water supplies. There are 1,233 public water supplies in the country, with 1.2 million consumers. The overall access to drinking water supply is 82.9%. The capital Tallinn has the highest access – up to 99%. In other towns the access is smaller and varies greatly in different towns and settlements.

The prevalence of small water supplies is a characteristic of Estonia. Eighty-six percent of water supplies serve up to 500 consumers and only 13 (1.1%) supplies have over 10,000 consumers. At the same time these bigger supplies serve a total of 65.5% of consumers.

Fluoride concentrations in Estonian groundwater vary significantly: from 0.01 up to 7.20 mg/L [7,8]. The distribution of fluoride concentration shows great regional differences within country.

High fluoride concentrations of natural origin are most typical in western Estonia, were analyzed fluoride contents reach up to 7.20 mg/L. This is the area where Silurian and Ordovician limestones and dolomites occur and the drinking water source is the Silurian-Ordovician aquifer system. Elevated fluoride concentrations can also be found along the northern outcrop line of Devonian rocks, where hydraulically connected Devonian and Silurian strata form the Devonian-Silurian aquifer system. Thus, permissible fluoride concentration set by drinking water standards are mostly exceeded in the Silurian-Ordovician carbonaceous aquifers, where approximately half of the analyzed fluoride contents are above 1.5 mg/L [8]. This is the reason why the most of the water supply systems with high fluoride content coincide with the outcrop of Silurian carbonate rocks (Figure 1).

The analysis of public water supplies providing high-fluoride water by number of consumers revealed that this is mainly a problem in small water supplies. In the majority (79%) of cases each water supply serves less than 500 inhabitants. Nevertheless, there are 11 (10.6%) supplies that serve each over 1,000 inhabitants. The biggest of them has 4,000 consumers. Generally, the smaller the water supply the higher was the mean fluoride content in the water supplied (Table 1).

Southern and north-eastern Estonia are characterized by a low fluoride content in the water and the population in northern Estonia consumes water with an optimal fluoride concentration. Low-fluoride groundwater is found in Devonian sedimentary rocks (Figure 1), where the major source of drinking water is the terrigenous Middle-Devonian aquifer system. The water supply in northern Estonia is based on the Cambrian-Vendian and Ordovician-Cambrian aquifer systems, which consist of sand- and siltstones that exhibit low or optimal fluoride values.

### 3.2. Exposure of Population to High Fluoride through Drinking Water

**Figure 2.**
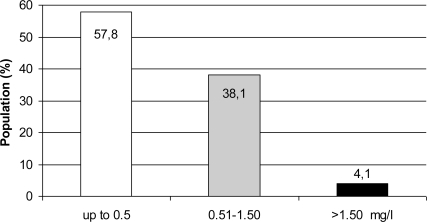
Distribution of population exposed to different fluoride levels in drinking water.

Figure 2 presents the general distribution of population exposed to different fluoride levels in Estonia. Low-fluoride water (up to 0.5 mg/L) is consumed by 57.8% of the population (607,544 inhabitants). Only 38.1% of the study population (400,040 inhabitants) was consuming water with medium fluoride concentration (0.51–1.50 mg/L). High-fluoride water (over 1.5 mg/L) is consumed by 4.1% (42,571 inhabitants) of the study population.

**Figure 3.**
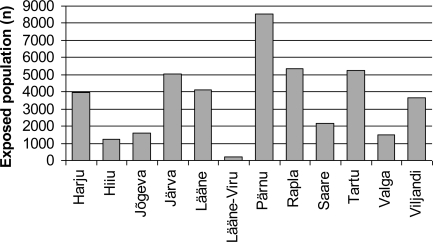
Distribution of population exposed to excessive fluoride levels (over 1.5 mg/L) by counties.

The situation differs to a large extent in different towns and counties. Excessive fluoride exposure was measured in twelve counties. In three counties (Ida-Viru, Põlva, Võru) the population was consuming water with fluoride content below 1.5 mg/L. Most of the population consuming high-fluoride water lives in Pärnu County, with others in Rapla, Tartu, Järva and Lääne counties (Figure 1, 3). The distribution of exposed population in four exposure categories and by counties is presented in Table 2. Over half of this population is exposed to slightly elevated levels of fluoride (group 1.51–2.0 mg/L).

Very high fluoride concentrations (over 4 mg/L) can be found only in western Estonia (Pärnu, Lääne, and Saare counties). Population exposed to highest values of fluoride is 5.7% of total risk group.

### 3.3. Risk of Dental Fluorosis in Relation to Drinking Water Fluoride Content

The original data of two studies on the prevalence of dental fluorosis were combined to investigate the quantitative relationship between dental fluorosis and drinking water fluoride content in the Estonian context. The prevalence of dental fluorosis among the study population (2,627) was 17.5%. In low-fluoride areas (<1.0 mg/L) the prevalence of dental fluorosis was very low (6.7%). Drinking water with higher fluoride level but still under maximum permissible limit (1.0–1.5 mg/L) doubled the prevalence of fluorosis. With the increase of fluoride levels in drinking water the dental fluorosis prevalence increased markedly (Table 3).

**Figure 4.**
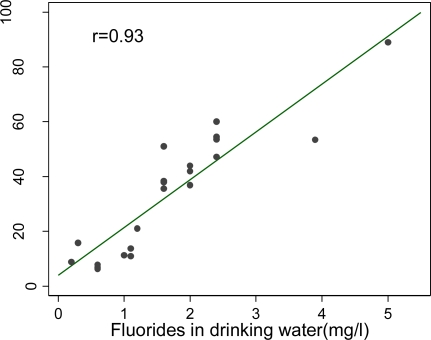
Correlation of dental fluorosis with drinking water fluoride.

The strong positive correlation (r=0.93) between drinking water fluoride content and the prevalence of dental fluorosis is illustrated in Figure 4.

The risk of dental fluorosis in case of excessive fluoride exposure was calculated for the four exposure categories (Table 4). The risk of disease was compared with risk in exposure category 1.0–1.5 mg/L fluorides in drinking water. The odds of developing dental fluorosis in exposure category 1.5–2.0 mg/L is 4.4 times higher compared to exposure below maximum permissible level of 1.5 mg/L. The risk is higher with the increase in fluoride levels in drinking water.

## 4. Discussion

This study was designed to evaluate Estonian population exposure to fluoride from drinking water. This study cannot comment on overall exposure to fluoride, although drinking water is usually the largest contributor to fluoride intake [15,16].

The study covered all 15 counties in Estonia and 93.7% of population having access to public water supplies. Although the fluoride content in groundwater is generally a stable parameter [17], changes to public supplies can lead to changes in fluoride concentrations in the water supplied. Our results showed good concordance with data from the Health Protection Inspectorate database and earlier studies in Estonia [6,18]. The population of Estonia has a high overall access to public water supplies (82.9%), but the variation between towns and rural settlements is large. The prevalence of a large number of small water supplies complicates the improvement of water quality.

Few studies on the occurrence and distribution of fluoride in groundwater have been carried out in Estonia [6,7,18]. Unfortunately, they have focused on a particular region or city. Quite often the tap water originates from different sources and is mixed in water supply systems. The result is that fluoride contents analyzed in raw ground or surface water does not represent what is in tap water. Our data from the analysis of drinking water samples collected throughout Estonia provide necessary information for assessment of human exposure to fluoride.

Based on current study, the great majority (about 96%) of Estonian population is drinking water with fluoride content below 1.5 mg/L. 4.1% of the population are exposed to high-fluoride drinking water in western Estonia (Lääne, Rapla and Pärnu counties), where the only drinking water source is Silurian-Ordovician aquifer system. The content of fluoride is related to the depth of the wells [7]. In these areas, there is no industry or any human activity that can cause anthropogenic contamination of the groundwater with fluoride and the high levels of fluoride are due to geogenic sources [19,20].

Elevated fluoride concentrations were also found in some water supplies of central Estonia (Tartu and Järva counties). This is the area where hydraulically connected Devonian and Silurian strata form the Devonian-Silurian aquifer system [19].

Endemic dental fluorosis is a common sight in many parts of the world with high fluoride drinking water. Our case study on Tartu showed there is up to six times higher risk of dental fluorosis among 12 year old school-children living in high fluoride (about 4 mg/L) region in comparison with children living in low-fluoride (about 0.2 mg/L) region [10].

In this study we were particularly interested in risk of population to fluoride concentration above 1.5 mg/L as this is the national maximum limit for fluoride. Therefore, the risk of disease was compared to risk in the group 1.0–1.5 mg/L. Fluorides in drinking water represent an avoidable excessive exposure. It is difficult and expensive to reduce a natural high level of fluoride in water. A drinking water standard should be met and there are number of means of doing. The first option should be to find an alternative source of water with suitable fluoride level. Surface water would be preferable. Also, mixing water from different sources can lower the fluoride level in drinking water (dilution with low-fluoride sources). If there is no other possible or cost-effective source, de-fluoridation of water must be attempted to avoid the toxic effects. The best solution depends on local circumstances. In the case of high fluoride level in drinking water the consumers should be informed and educated about their potential risk, giving them advices to optimize their intake of fluoride. For example, bottled water would be recommended for drinking and food processing purposes. However, this recommendation is only a temporary solution in extreme cases and is not sustainable.

## 5. Conclusions

This study provides an overview of the fluoride content in drinking water and the extent of human exposure to different levels of fluoride through drinking water for the whole of Estonia. Exposure analysis revealed that majority of people (over 90%) consumed water with fluoride content below the maximum permitted level (1.5 mg/L) and over half of the study population was exposed to water containing very low fluoride levels. Very high naturally occurring fluoride concentrations (up to 7 mg/L) are common in western Estonia, where the drinking water source is the Silurian-Ordovician aquifer system. It would be important to determine the health problems that might be caused by excess fluoride intake in this region.

The study supports previous knowledge on positive relationship between natural fluoride in drinking water and dental fluorosis. The study showed that fluoride concentration over 1.5 mg/L poses high risk to people. The odds to develop dental fluorosis are 4.4 times higher in regions with water fluoride content 1.5–2.0 mg/L. The risk increases with the increase of fluoride content in drinking water.

As drinking water is usually the main source of fluorides intake, the levels of fluoride in tap water is very important in determining total fluoride exposure. The information of population about the levels of fluoride in drinking water is an important key element in health protection. Adequate information and recommendations help people themselves to make informed decisions and regulate health risks from drinking water. The results of the current study as well as other available data from water quality should be taken into account when developing strategies for safe drinking water supplies.

Strategic plans and public awareness campaigns on fluorides in drinking water and health impact should be implemented.

## Figures and Tables

**Table 1.** Distribution of high-fluoride water supplies by number of consumers (size).

Size of water supplies (consumers served)	Water supplies		Concentration of fluoride (mg/L)	

	n	%	Mean	SD
1,001–5,000	11	10.6	2.11	0.92
501–1,000	11	10.6	2.50	0.95
101–500	67	64.4	2.69	1.09
up to 100	15	14.4	2.99	1.39
Total	104	100	2.57	1.12

**Table 2.** Population exposure to excessive fluoride in drinking water by counties.

County	Numbers of population exposed to excessive fluoride by exposure categories (mg/L)				
	1.51–2.0	2.1–3.0	3.1–4.0	> 4.0	Total
Harju	3,978	0	0	0	3,978
Hiiu	1,228	0	0	0	1,228
Ida-Viru	0	0	0	0	0
Jõgeva	580	691	300	0	1,571
Järva	4,696	200	130	0	5,026
Lääne	190	1,540	1,640	740	4,110
Lääne-Viru	225	0	0	0	225
Pärnu	1,919	1,266	4,117	1,260	8,562
Rapla	2,890	2,158	306	0	5,354
Saare	1,030	400	260	450	2,140
Tartu	3,472	1,400	350	0	5,222
Valga	1,500	0	0	0	1,500
Viljandi	1,313	2,342	0	0	3,655

Total	23,021	9,997	7,103	2,450	42,571

**Table 3.** Prevalence of dental fluorosis in different fluoride levels in drinking water.

Fluoride content mg/L	No of children	Children with fluorosis (cases)	Healthy subjects (controls)	Prevalence of fluorosis (%)
<1.0	1,024	69	955	6.7
1.0 – 1.5	984	120	864	12,2
1.51 – 2.0	386	147	239	38.1
2.1 – 3.0	167	75	92	44.9
3.1 – 4.0	30	16	14	53.3
>4.0	36	32	4	88.9

Total	2,627	459	2,168	17.5

**Table 4.** The risk of dental fluorosis in relation to different fluoride exposure.

Exposure category	Odds ratio (OR)	Confidence interval (CI)
1.51–2.0 mg/L	4.4	3.3 – 5.9
2.1–3.0 mg/L	5.9	4.1 – 8.4
3.1–4.0 mg/L	8.2	3.9 – 17.3
> 4 mg/L	57.6	20.0 – 165.7

## References

[1] WHO (2004). Guidelines for drinking-water quality.

[2] Ministry of Social Affairs Joogivee kvaliteedi- ja kontrollinõuded ning analüüsimeetodid. [The quality and monitoring requirements for drinking water and methods of analysis] Riigi teataja 2001 100 1369(in Estonian).

[3] Fawell J, Bailey K, Chilton J, Dahi E, Fewtrell L, Magara Y (2006). Fluoride in drinking-water.

[4] Alarcon-Herrera MT, Martin-Dominquez I, Trejo-Vazquez R, Rodriquez-Dozal S (2001). Well water fluoride, dental fluorosis, bone fractures in the Guadiana Valley of Mexico. Fluoride.

[5] Kurttio P, Gustavsson N (1999). Exposure to natural fluoride in well water and hip fracture: A cohort analysis in Finland. Am. J. Epidemiol.

[6] Saava A Uibo M Ratnik V Mikroelementide sisaldus Eesti vetes ja nende osa kohalikus patoloogias [Content of microelements in water in Estonia and their role in local pathology] Eesti Loodus 1973 10 606 608(in Estonian).

[7] Karro E, Rosentau A (2005). Fluoride levels in the Silurian-Ordovician aquifer system of western Estonia. Fluoride.

[8] Karro E, Indermitte E, Saava A, Haamer K, Marandi A (2006). Fluoride occurrence in publicly supplied drinking water in Estonia. Environ. Geol.

[9] Kiik V Joogivee erineva fluorisisalduse mõjust laste hammaskonna seisundile Eesti NSV tingimustes. [The influence of different fluoride content in drinking water to dental status of children in Estonian SSR]Doctoral thesis; Research Institute of Epidemiology, Microbiology and Hygiene Tallinn, Estonia 1970(in Estonian).

[10] Indermitte E, Russak S, Saava A (2005). The contribution of drinking water towards dental fluorosis: A case study in Tartu. Paper Anthropol.

[11] Indermitte E, Karro E, Saava A (2007). Tap water fluoride levels in Estonia. Fluoride.

[12] Dean HT (1934). Classification of mottled enamel diagnosis. J. Am. Dent. Assoc.

[13] WHO (1997). Oral Health Surveys Basic Methods.

[14] Cameron AC, Widmer RP (1997). Handbook of pediatric dentistry.

[15] Zohouri F, Rugg-Gunn AJ (2000). Sources of dietary fluoride intake in 4-year-old children residing in low, medium and high fluoride areas in Iran. Int. J. Food Sci. Nutr.

[16] Erdal S, Buchanan SN (2005). A quantitative look at fluorosis, fluoride exposure, and intake in children using a health risk assessment approach. Environ. Health Persp.

[17] Shomar B, Müller G, Yahya A, Askar S, Sansur R (2004). Fluorides in groundwater, soil and infused black tea and the occurrence of dental fluorosis among school children of the Gaza Strip. J. Water Health.

[18] Kuik L Joogivee fluori- ja joodisisaldus Eesti NSV-s [Fluoride and iodine content of drinking water in Estonian SSR] Kurortoloogilised Uurimised 1963 2 39 45(in Estonian).

[19] Karise V Metsur M Perens R Savitskaja L Tamm I Eesti põhjavee kasutamine ja kaitse. [Use and protection of groundwater in Estonia] Eesti Põhjaveekomisjon Tallinn, Estonia 2004(in Estonian).

[20] Haamer K, Karro E (2006). High fluoride content of K-bentonite beds in Estonian Paleozoic carbonate rocks. Fluoride.

